# The Association Between Childhood Leukemia and Population Mixing: An Artifact of Focusing on Clusters?

**DOI:** 10.1097/EDE.0000000000000921

**Published:** 2018-11-30

**Authors:** Laurie Berrie, George T.H. Ellison, Paul D. Norman, Paul D. Baxter, Richard G. Feltbower, Peter W.G. Tennant, Mark S. Gilthorpe

**Affiliations:** From the a School of Medicine, University of Leeds, Leeds, LS2 9NL, UK; b Leeds Institute for Data Analytics, University of Leeds, Leeds, LS2 9NL, UK; c School of Geography, University of Leeds, Leeds, LS2 9JT, UK.

**Keywords:** Population mixing, Childhood leukemia, Simulation, Health geography

## Abstract

**Background:**

Studies investigating the population-mixing hypothesis in childhood leukemia principally use two analytical approaches: (1) nonrandom selection of areas according to specific characteristics, followed by comparisons of their incidence of childhood leukemia with that expected based on the national average; and (2) regression analyses of region-wide data to identify characteristics associated with the incidence of childhood leukemia. These approaches have generated contradictory results. We compare these approaches using observed and simulated data.

**Methods:**

We generated 10,000 simulated regions using the correlation structure and distributions from a United Kingdom dataset. We simulated cases using a Poisson distribution with the incidence rate set to the national average assuming the null hypothesis that only population size drives the number of cases. Selection of areas within each simulated region was based on characteristics considered responsible for elevated infection rates (population density and inward migration) and/or elevated leukemia rates. We calculated effect estimates for 10,000 simulations and compared results to corresponding observed data analyses.

**Results:**

When the selection of areas for analysis is based on apparent clusters of childhood leukemia, biased assessments occur; the estimated 5-year incidence of childhood leukemia ranged between zero and eight per 10,000 children in contrast to the simulated two cases per 10,000 children, similar to the observed data. Performing analyses on region-wide data avoids these biases.

**Conclusions:**

Studies using nonrandom selection to investigate the association between childhood leukemia and population mixing are likely to have generated biased findings. Future studies can avoid such bias using a region-wide analytical strategy. See video abstract at, http://links.lww.com/EDE/B431.

## Abstract 2

Supplemental Digital Content is available in the text.

When comparing disease incidence between areas, those areas with small populations are more likely to appear as spatial clusters of high incidence by chance alone. Focusing on these supposed clusters is therefore a poor basis on which to generate or test causal hypotheses.^1^ Nonetheless, such clusters are hard to ignore^2^ and can generate substantial pressure for plausible explanations. This may explain the considerable public and political interest given to the high incidence of childhood leukemia in Seascale (Cumbria, UK) during 1963–1983 and the relative lack of attention to the absence of such cases during 1991–2006.^3,4^

The challenges of examining clusters between areas with different population sizes are likely to have influenced the development and testing of the “population mixing hypothesis”. The idea emerged from analyses purporting to show an association between “population mixing” and childhood leukemia, interpreted as evidence for the involvement of infectious agents. The hypothesis proposes that the immune systems of children resident in more isolated and/or less densely populated communities are more likely to have been exposed to a less diverse range of infectious agents than residents in less isolated and/or more densely populated communities. These children are therefore believed to be more likely to develop leukemia once exposed to novel infections from inward migrants.^5^

This hypothesis is both persuasive^6^ and enduring^7^ but relies on several untested assumptions and involves a lack of clarity around how many of its key concepts should be defined, measured, and analyzed.^8^ One assumption is that isolated communities, and those with lower population densities, are less likely to experience the frequency/intensity of contact required to sustain infections. Another is that communities with lower rates of inward migration are less frequently exposed to exogenous infections. While these assumptions reflect established tenets of infectious disease epidemiology, they require levels of isolation, population dispersion, and (im)mobility that remain unspecified and may be neither plausible nor applicable where the hypothesis has been examined. There also remains extensive disagreement regarding the roles that the immune system and early exposures to infection play in the etiology of childhood leukemia.^9–11^

These mechanistic uncertainties are compounded by a lack of consensus concerning: what constitutes an isolated or less dense population; criteria used to distinguish between migrants and residents; and how these concepts are operationalized as measures of population mixing. Researchers exploring the association between population mixing and childhood leukemia have therefore used a range of different measures as proxies for population mixing including differences and/or changes in population size/density; the proportion and/or diversity of inward-migrants; and versions of the Shannon Diversity index.^8^

The variety of measures confirms a lack of conceptual precision/consensus and reflects the practical constraints imposed by the distribution and migration patterns of populations within regions where suitable data exist; the collation/organization of data on these parameters; and challenges differentiating leukemia cases among residents and inward-migrants. Good quality, area-level data on population size/density, migration, and childhood leukemia incidence are only available for high-/middle-income countries where large regions are usually subdivided into small areas along political/administrative rather than demographic lines. These small areas display substantial variation in geospatial features (size, shape, and distance apart) and in the size/distribution of their constituent populations. Consequently, along with the sociodemographic detail of data available from sources such as a decennial census, the geographical specification of these areas constrains what measures of isolation, density, migration, and mixing can be generated. Such subdivision also creates larger-than-expected chance variations in incidence among smaller populations simply due to chance.^12^

Different researchers have used different analytic strategies, generating contradictory results.^5,13^ Some of the earliest studies followed the identification of an apparent cluster of leukemia cases in a single area and sought to verify whether this constituted a bona fide cluster (i.e., a higher number of cases than expected given the national/regional incidence proportion—the number of new cases per population at risk during a particular period of time).^14^ Unfortunately, such studies provide little evidence of whether the elevated incidence is associated with any characteristics of the area concerned. In these studies, it is often unclear how/when the specific measures for population mixing were selected (i.e., *before* or *after* the areas of study were selected for their apparent excess of cases). Substantial methodologic variations make it challenging to identify commonalities in analytical approach for closer examination. However, many such studies focused specifically on areas displaying childhood leukemia clusters/higher incidence of childhood leukemia. Indeed, where other studies adopted a nonselective region-wide analytic strategy—examining associations between area-based measures of population mixing and leukemia incidence across the whole region or in a random sample of areas—these tend to generate contradictory findings to those adopting nonrandom, selective, or focused analytic strategies.^5,14–20^

Much work is needed to strengthen the concepts, measures, and datasets used to test the population mixing hypothesis. There is a pressing need to establish why different analytic strategies generate such contradictory findings. This study uses simulation and analysis of observed data to examine the two principal analytic strategies used by previous ecological studies and explores the relationship between commonly used measures of population mixing and childhood leukemia. Such measures typically draw on the concept of population mixing as proposed by the first study to use this term,^5^ which was subsequently defined as an “increase in population density produced by a marked influx into a rural area” (where “rural” was considered a less densely populated area).^21^ On this basis, we chose the two most common measures of population mixing used by previous studies: population density and inward migration. Population density provides a measure of the number of individuals capable of spreading a putative leukemia-promoting infectious agent,^8,22^ expressed as the population per unit area. Inward migration provides a measure of the relative number of new arrivals capable of bringing such agents with them, expressed as the proportion of migrants within the population. We calculated both measures using existing data disaggregated by administrative areas and used them to undertake each of the analytic strategies as follows:

1. Selective subregion analysis. We selected areas with contrasting values of population density, inward migration, and/or childhood leukemia incidence (i.e., representing areas of specific interest as potentially highly exposed versus reference areas) nonrandomly for direct comparison.
2. Region-wide analysis. The relationship between population density, inward migration, and childhood leukemia incidence is examined using standard regression techniques across all small areas within a larger region or a random sample of areas.

## METHODS

We applied selective subregion analysis and region-wide analysis to observed data from the Yorkshire and Humber region of the United Kingdom using data from a previous study of the population mixing hypothesis.^13^ We also simulated data in which the number of childhood leukemia cases was determined solely by population size and not by population density or inward migration (i.e., the null hypothesis). We used the statistical software package R throughout.^23^

### Observed Data

We calculated population density and inward migration for each of the 532 census wards in the Yorkshire and Humber region using 1991 census data on total population; ward area (km^2^); number of inward migrants (those with a different address one year prior to the census); and number of 0–14 year olds (the population we considered at risk). We calculated population density prior to inward migration. We calculated inward migration in relation to each ward’s premigration population, such that the proportion of inward migration could exceed one (i.e., for wards where inward migration resulted in a doubling, or more, of the population).

**Figure 1.**
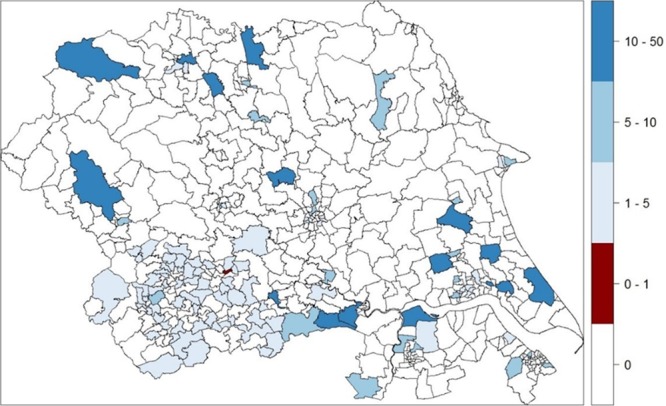
Ratio of observed to expected (based on average national incidence) cases of childhood leukemia in Yorkshire and Humber (United Kingdom), 1988–1993, by ward.

We identified leukemia cases (for 0–14 year olds) from the Yorkshire Specialist Register of Cancer in Children and Young People, diagnosed within the Yorkshire Regional Health Authority between 1988 and 1993 (the closest date to the 1991 census for which data were available).^13^ These were mapped to census wards to permit estimation of childhood leukemia incidence rates (Figure 1). Situating these analyses around the 1991 census facilitated comparison with previously published studies, most using data before subsequent declines in incidence reported elsewhere.^3,4^

### Simulated Data

We simulated multivariate ward-level data on population density and inward migration such that their distributions and correlation structure approximated those in the observed data.^24^ We based simulated cases on the national childhood leukemia incidence proportion over a comparable 5-year period^25^; a time interval chosen to emulate previous studies and overcome key challenges with modeling rare events. Simulations used the Poisson distribution (i.e., as evident in cases of childhood leukemia in the observed data) under the null hypothesis that the number of cases of childhood leukemia in each area is determined only by the number of 0- to 14-year-olds (see eAppendix 1; http://links.lww.com/EDE/B411 for a step-by-step guide to the simulation). By approximating the observed population structure under the null assumption that the only driver of the number of cases of childhood leukemia is population size, deviations from a null result in the analyses of simulated data must be due to selection or analytic errors. To ensure sufficient data were available to reduce the standard error of the simulation process^26^ and to more precisely learn the operating characteristics of the different estimation procedures, we generated 10,000 simulated datasets.

### Selective Subregion Analytic Strategy

To emulate the selective subregion strategy, we selected 16 wards, the mean number of areas in those studies that used this approach,^5,14–20,27^ based on extreme values of low population density, high inward migration, high childhood leukemia incidence, or combinations of all three. We examined 15 selection scenarios (based on all combinations of these three selection variables) to account for the disparate methods found in the literature.

Scenarios 1–3 involved ranking wards according to low population density, high inward migration, or high incidence alone, then randomly selecting 16 of the highest ranked 50% of wards for analysis. Scenarios 4–9 involved ranking wards according to each possible pair of variables: ranking first on the initial variable and selecting the highest 50%, next ranking these on the second variable and selecting the highest 50%, then randomly selecting 16 wards for analysis. Finally, Scenarios 10–15 involved (1) ranking the wards according to every possible ordering of all three variables—ranking on the initial variable and selecting the highest 50%; (2) on the second variable, selecting the highest 50%; (3) on the third variable, again selecting the highest 50%, before (4) randomly selecting 16 wards for analysis. To match the number of random selections available from the 10,000 simulated datasets, we also took the random selection of the 16 wards 10,000 times on the observed data.

For each of these 15 scenarios, we reported median values of the estimated childhood leukemia incidence with their empirically derived 95% ranges (95% range: 2.5% and 97.5% estimates from the 10,000 datasets). We aggregated figures from the 16 selected wards and compared the total number of cases observed with the number expected from the national incidence in people aged 0 to 14 years using the binomial exact test.^28^ The proportion of significant *P* values (5% level) for each test, together with the direction of the corresponding estimates (above/below the national incidence rate), was recorded. For simulated data, the proportion of significant *P* values (5% level) is equivalent to the estimated type I error rate. *P* values have been included along with confidence intervals as the original studies reported these.

### Region-wide Analytical Strategy

To replicate the region-wide strategy of previous studies,^13,29–36^ we used Poisson regression models to match the distribution evident in the observed data and that used in the generation of the simulated datasets. Three separate regression models were conducted on a random selection of 50% of wards using population density or inward migration, or both as covariates (corresponding to Scenarios 1, 2, 4, and 5 of the selective subregion analytical strategy, above). The arbitrary choice of selecting a random sample of 50% of the data for analysis was to ensure that the impact of random sampling variation across the simulations was present in both region-wide and selective subregion approaches. Each model was generated 10,000 times for the observed data to facilitate comparisons with analysis of the 10,000 simulated datasets. Median risk ratios and their empirically derived 95% ranges (95% range: 2.5 and 97.5 centile estimates from the 10,000 datasets) are described for a 25% increase in population due to inward migration and for a population density increase of 500 persons per square kilometer. Since population density is a continuous variable, a contrast between two states cannot be easily described; instead, the effect of an absolute increase in population density is reported. The *P* values corresponding to each risk ratio were recorded, combined with whether the risk ratio was above (harmful effect) or below (protective effect) one.

### A Note on P Values

We believe that null hypothesis significance tests are inappropriate for observational data analyses, and we would not typically use them. Unfortunately, they remain extremely common in the wider literature, and all the historical studies that we are emulating used null hypothesis significance tests based on *P* value thresholds. For comparison to these previous studies, we explore the results in terms of the likelihood of obtaining *P* < 0.05, in addition to (our preferred) absolute effect size.

## RESULTS

### Selective Subregion Analytic Strategy

Analyses of 10,000 random samples drawn from the observed dataset using each selective subregion scenario (Table 1) indicate that where selection was based on *low* population density or *high* inward migration alone or both (Scenarios 1, 2, 4, and 5), the proportions of significant *P* values were low (ranging from 1.3% to 3.6%). Where selection was based on either a *high* incidence of leukemia, either alone or together with one or both exposures (Scenarios 3 and 6–15), the proportions of significant *P* values were substantially greater than the 5% that would be expected if the null were true (ranging from 18.4% to 97.2%).

**Figure 2.**
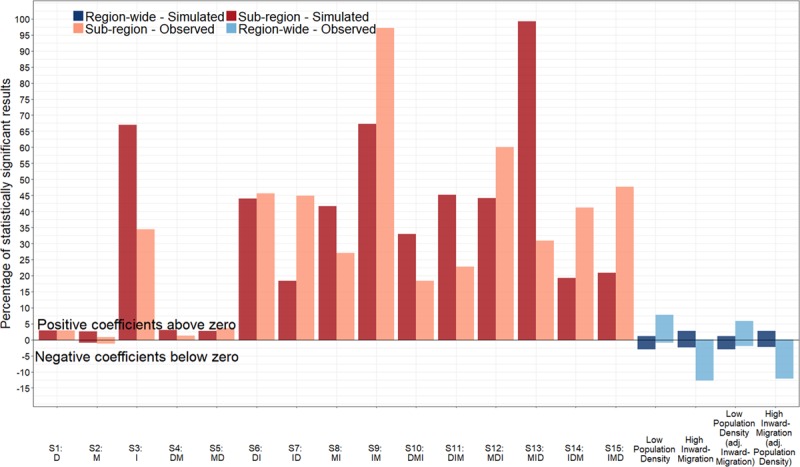
Percentage of statistically significant results at the 5% level by analytical strategy for both simulated and observed data. Selective subregion analytical strategy results were analyzed using the binomial exact test; direction of the bars indicates whether the estimated probabilities of the significant test results were greater (above zero) or less than (below zero) the national average. Region-wide analytical strategy results were analyzed using Poisson regression; direction of the bars indicates whether statistically significant coefficients were greater (above zero) or less than (below zero) zero. D, population density; M, inward migration; and I, childhood leukemia incidence; order of letters indicates the order used to select data for analysis.

For analyses of data simulated under the null hypothesis, type I error rates of 2.8% to 3.7% were observed under Scenarios 1, 2, 4, and 5 (Table 1), consistent with random subregion selection (i.e., 3.5% type I error rate). Where selections were based on a *high* incidence of leukemia either alone or together with one or both exposures (Scenarios 3 and 6–15), type I error rates were far higher (ranging from 18.4% to 99.3%; Figure 2).

**TABLE 1.**
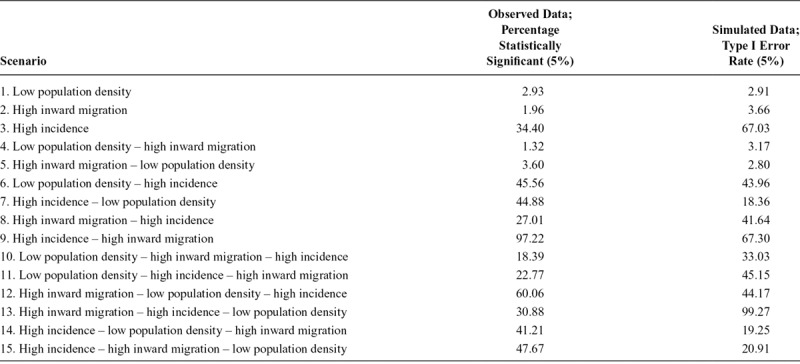
Type I Error Rates of the Selective Subregion Analytical Strategy Under Each of the Scenarios Examined

**Figure 3.**
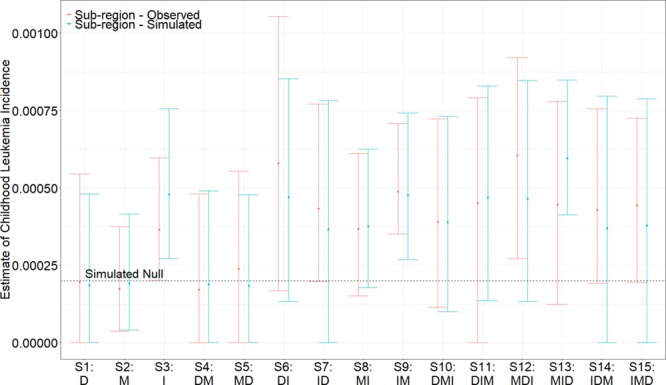
95% empirically derived ranges (95% range: 2.5 and 97.5% centile estimates from the 10,000 datasets, points indicate the median) of the distribution of childhood leukemia incidence from the binomial exact test of the selective subregion analytical strategy. The dashed line indicates the incidence rate used to generate the simulated datasets, that is, two cases per 10,000 zero- to fourteen-year olds in a 5-year period. This is the incidence expected under the null hypothesis; any deviation from this indicates bias. D, population density; M, inward migration; and I, childhood leukemia incidence; order of letters indicates the order used to select data for analysis.

The estimated 5-year incidence of childhood leukemia ranged between zero per 10,000 and eight per 10,000 children across the 10,000 simulated datasets, indicating that up to eight cases per 10,000 children might occur by chance in any 5-year period. This is in contrast to what was simulated, that is, two cases per 10,000 population. The range of estimates were similar in the observed datasets (Figure 3).

### Region-wide Analytic Strategy

The proportions of significant *P* values in region-wide analyses of observed data all exceeded 5% (7.9%–13.0%; Table 2), suggesting that high inward migration was associated with lower childhood leukemia incidence and *low* population density was associated with a higher childhood leukemia incidence.

**TABLE 2.**
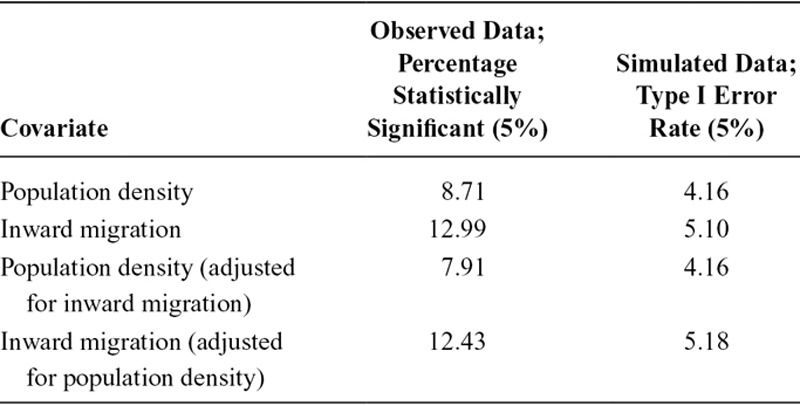
Type I Error Rates of the Region-wide Analytical Strategy According to the Covariate Examined in the Model

Region-wide analyses of simulated data returned type I error rates between 4.2% and 5.2% for all model coefficients (Table 2). The distribution of the coefficient values is not centered on zero (i.e. −-2.5% to 2.5%) due to small, but non-zero, correlations between cases of childhood leukemia, population density, and inward migration, which arise from a mathematical dependency between these variables (see Supplemental Digital Content, eFigure S1; http://links.lww.com/EDE/B411).

In the simulated data, the median risk ratios (RR) for the effects of inward migration were consistently 1.0, indicating agreement with the null hypotheses (e.g., RR vs 0% migration: 25% = 1.0 [95% range = 0.08–8.81]). In the observed data, however, increasing levels of inward migration were associated with lower incidence of leukemia (e.g., RR vs 0%: 25% = 0.33 [95% range = 0.02–2.05]).

**Figure 4.**
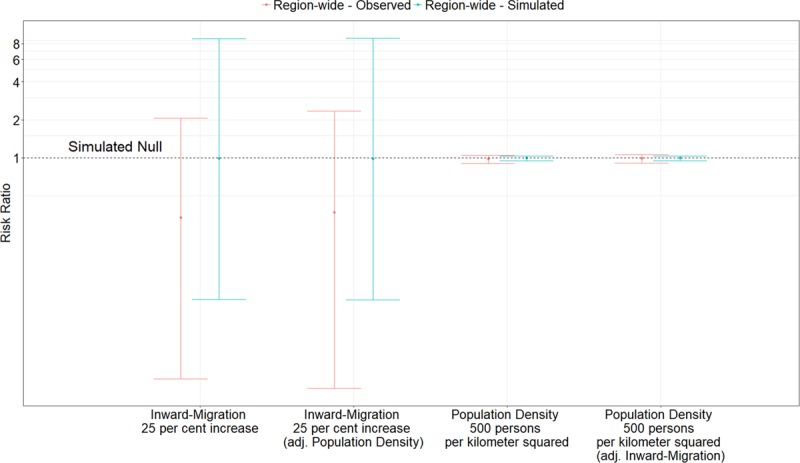
95% empirically derived ranges (95% range: 2.5 and 97.5% centile estimates from the 10,000 datasets, points indicate the median) of the percentage increase or decrease in childhood leukemia incidence from the regression models of the region-wide analytical strategy with an increase of inward migration of 25% and an increase in population density to 500 persons/km^2^. The dashed line indicates no change in childhood leukemia incidence as expected under the null hypothesis. Results shown with log scaling.

All risk ratios for the effect of population density in both the simulated data and observed data were close to 1.0, indicating consistent agreement with the null hypotheses (RRs per unit increase in person/km^2^ in simulated data: 500 people/km^2^ = 1.0 [95% range = 0.95–1.03]; in observed data: 500 people/km^2^ = 0.98 [95% range = 0.90–1.05]). Coefficients of adjusted regression models (including both inward migration and population density as covariates) did not materially differ from those in unadjusted models (Figure 4).

## DISCUSSION

The present study uses simulation and observed data analysis to contrast the two most commonly used analytical strategies found in the literature that investigate the proposed relationship between population mixing and childhood leukemia incidence. We demonstrate how the different analytical strategies used to examine the relationship between “population mixing” and childhood leukemia incidence can generate radically divergent results. Considerable bias occurs if geographic areas are selected prior to analysis and selection is influenced by elevated childhood leukemia incidence (i.e., clusters). Bias is also evident where selection involves measures of population mixing, as population mixing appears adversely associated with elevated childhood leukemia incidence.

We did not examine all the measures of population mixing used by previous studies and did not attempt to generate alternative proxies for population mixing. Our aim was to examine the impact of the two most common analytical strategies using two typical measures of population mixing. Furthermore, since the observed and simulated data did not differentiate between cases of childhood leukemia among residents and inward migrants, we could not assess whether population mixing might be associated with a differential risk of childhood leukemia in each. However, the small numbers of inward migrants, and of 0- to 14-year-olds therein, would make such analyses challenging and may explain why few previous studies have sought to do this. A further limitation of the present study is that we did not examine possible temporal effects related to the timing of population mixing events and/or age at exposure. Despite substantial variation in these criteria among previously published studies, few sought to examine their impact on the direction or strength of the associations found. This, however, is less relevant to our study’s focus on the comparison of analytic strategies.

Analyses of rare diseases such as childhood leukemia are challenging because disease registries often only collect summary information on the denominator (or population at risk) within areas for aggregated blocks of time. By far, the most common type of analysis is therefore to conduct aggregated analyses of incidence proportions, that is, comparing cases per population within fixed units of time. Because of the size of the areas typically examined, and the rarity of childhood leukemia, such analyses are prone to an abundance of zero cell counts. The most common solution is to preserve the area level granularity and collapse the time frame into longer periods, with 5-year periods being the most common approach in the literature; we have adopted the same approach in our indicative analyses. Alongside the limitations imposed from using data from disease registries mentioned above, we are also limited to describing averaged area-level migration patterns from census data which measure migration based on change of address from a year prior to the census date. Therefore, we cannot take account of the likely time lag between exposure and event; however, this is also a limitation of many of the studies we wish to emulate.

Notwithstanding these limitations, our study confirms that analyses based on nonrandom area/subregion selections that are influenced by or associated with elevated childhood leukemia incidence can generate entirely erroneous findings. In all scenarios with such selection, associations were profoundly biased, falsely suggesting that low population density and/or high inward migration were associated with elevated childhood leukemia incidence.

Unfortunately, the lack of methodologic clarity in research adopting a selective subregion analytical strategy means it is not possible to establish which studies might be prone to biases associated with this strategy. Even if studies sought to select areas using only variables chosen as measures for population mixing, it is feasible that selection was affected instrumentally (by co-dependence on demographic characteristics) or implicitly (by knowledge of, interest in, or attention to the outcome). The latter is likely to be central to the importance afforded to clusters of similarly rare events. It seems likely that focusing on clusters of childhood leukemia, together with the confirmatory results produced by selective subregion analyses, researchers are encouraged to use this analytic strategy, unaware of the bias it generates. This would explain the publication bias among studies examining the population mixing hypothesis.^21^ Studies using the unbiased region-wide approach are more challenging to publish because they fail to identify the large artifact found in selective subregion analyses. Nevertheless, region-wide analytic strategies avoid the risk of explicit or implicit attention to clusters, ensure that selection biases cannot occur, and ensure the analysis can be extended to cover any available geographic characteristics. For this reason, ecologic studies of the population-mixing hypothesis that have used a nonrandom selective subregion approach should be viewed with extreme caution.

## CONCLUSIONS

Future studies investigating the association between population mixing and childhood leukemia (or other apparently clustered events) should adopt a region-wide analytic strategy to avoid the potential biases inherent in a nonrandom selective subregion approach. Where an entire dataset is not available for analysis, sampling should be random to avoid potential subregion selection biases. Syntheses of previous studies examining this association should place greater emphasis on findings from studies adopting region-wide analyses and only consider findings from those studies using selective subregion analyses where the authors have explicitly used random selection methods to avoid the potential risk of focusing on areas exhibiting apparent clusters (i.e., a high incidence) of leukemia.

## Supplementary Material
