# Supplementary material for: The Association Between Childhood Leukemia and Population Mixing: An Artifact of Focusing on Clusters?
Source: Epidemiology. 2018 Nov 30;30(1):75–82. doi: 10.1097/EDE.0000000000000921 (PMC6276864; doi:10.1097/EDE.0000000000000921)
Supplement: Supplementary file 1 [file ede-30-075-s001.pdf]

Supplemental Digital Content

Contents

Principles of simulating the data Page 1
Causal Structure Page 3
Appendix References Page 4

Principles of simulating the data

For the simulations that formed the basis of this research, exploratory data analyses were conducted on data from the Yorkshire and Humber region, which included fitting approximate distributions to four variables using the **R** function fitdistr from the MASS package[1]: the total population; the population of 0-14 year olds; the ward area (km^2); and total inward-migration (eTable 1). These distributions were passed to the GenData **R** function,[2] along with the correlation structure (eTable 2) of the Yorkshire and Humber data, to simulate four new variables for 532 electoral wards (the same number as in the Yorkshire and Humber region). This function generates multivariate non-normal data that has an approximate correlation structure as specified by the user. The number of leukaemia cases per geographical area are not simulated in this step.

Where simulation generated negative/implausibly small values (determined by whether they were smaller than those in the observed dataset), these were replaced by random values between the minimum and median observed values in the observed dataset (Random Draw Limits in eTable 1).

Leukaemia case data were generated using a Poisson distribution across all 532 electoral wards based solely on the population of 0-14 year olds of each electoral ward generated from the GenData **R** function (note: this assumes the null hypothesis that all measures of population mixing have no effect on leukaemia incidence).

eTable 1: Distributions upon which simulated variables were based, and the limits between which values were drawn to replace infeasible/unrealistic values.

Variable	Distribution	Random Draw Limits
Total population:	NB[a](mu = 6500, theta = 2.0)	450, 6000
0-14 year old population:	NB[a](mu = 1300, theta = 1.6)	70, 1300
Area size (km^2):	NB[a](mu = 26, theta = 0.7)	0.17, 16
Number of inward-migrants:	NB[a](mu = 500, theta = 1.8)	450, 3600
Cases of childhood leukaemia:	P[b](lambda = 0-14 year old ward population*0.0002)	-

[a]NB = Negative-binomial distribution;

[b]P = Poisson distribution.

eTable 2: Correlation matrix used as input for the GenData function[2], calculated from the observed data from Yorkshire and Humberside

	Population (0-14)	Area	Inward-Migrants	Total Population
Population (0-14)	1.000	-0.296	0.895	0.971
Area	-0.296	1.000	-0.323	-0.293
Inward-Migrants	0.895	-0.323	1.000	0.918
Total Population	0.971	-0.293	0.918	1.000

To reduce the possibility of correlation between each of the generated datasets, the starting seed for each simulation was separated by the number of wards in each dataset and the number of variables that were being generated (i.e. 532 x 4 = 2128).[3]

The chosen population mixing proxies "proportion of inward-migration" and "population density" were calculated from the raw simulated variables, i.e. proportion of inward-migration = number of inward-migrants/total population and population density = total population/area size (km^2).

For the sub-region strategy, 16 wards were selected according to the fifteen selection scenarios introduced in the main text. The 16 wards are aggregated and the binomial exact test is performed on each of the aggregated totals.

For the region-wide strategy, half of the data is randomly selected for analysis (this is done to mimic random selection that may be done in region-wide studies and to highlight that if the sampling is truly random it has no impact on the results of region-wide studies).

This is repeated 10000 times, i.e. 10000 simulated datasets are generated and the two methods are applied to each simulated dataset. The results of the tests are recorded and reported in the main text. **R** code is available on GitHub (via the primary author's account), code for the observed data analysis is omitted as the observed data is not publicly available.

Causal structure

The demographic data were simulated such that the correlation structure equals that of the observed data for the Yorkshire and Humber region as per the causal structure depicted below: i.e. under the null hypothesis, only the population size causally influences the number of childhood leukaemia cases and there is no causal arrow between inward-migration and the size of the area. There will be a non-zero correlation between the number of ‘Cases’ and all four area measures (‘Area Size’, ‘Population Density’, number of ‘Inward-Migrants’, and the ‘Proportion of Inward-Migrants’: Figure A1) because ‘Population’ is causally related to them all. Since ‘Population’ is an offset term in the Poisson regression model, conditional independence between ‘Cases’ and both ‘Area Size’ and the number of ‘Inward-Migrants’ is assured due to ‘controlling’ for ‘Population’. Conditional independence is not achieved between ‘Cases’ and either derived ratio variable (‘Population Density’ and ‘Proportion of Inward-Migrants’) by ‘controlling’ for the ‘Population’ offset because both derived ratio variables contain an element of ‘Population’ explicitly; this explains a lack of symmetry in some of the p-values in Figure 2.

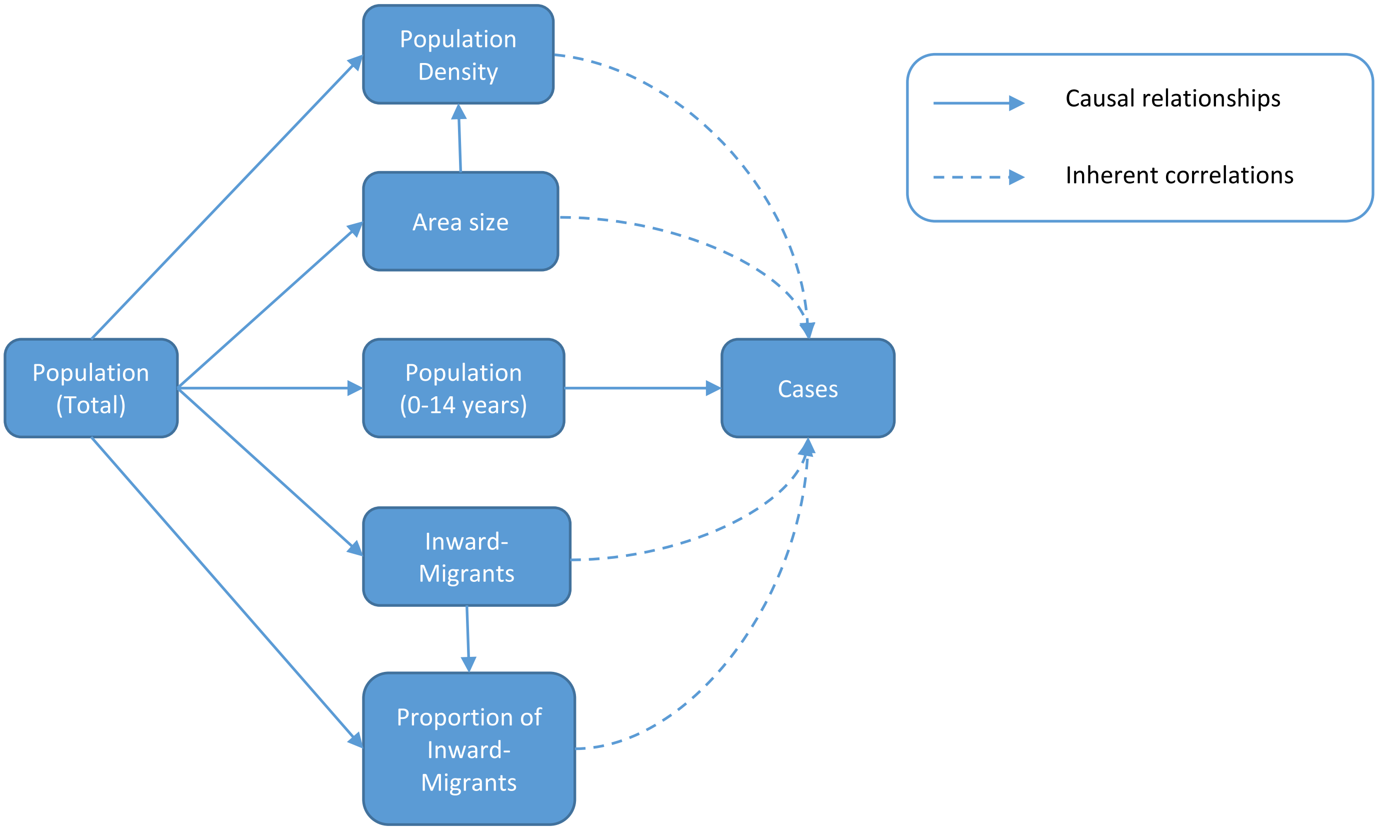

eFigure S1: Graph representing the simulated relationships of variables within the dataset: causal relationships are represented by solid arrows and implicit correlations are represented by dashed arrows.

Appendix References

1. Venables WN, Ripley BD. (2002) Modern Applied Statistics with S. Fourth Edition. Springer. New York. ISBN 0-387-95457-0

2. Ruscio J, Kaczetow W. Simulating Multivariate Nonnormal Data Using an Iterative Algorithm. Multivariate Behav Res. 2008;43(3):355-381. doi:10.1080/00273170802285693.

3. Burton A, Altman DG, Royston P, Holder RL. The design of simulation studies in medical statistics. 2006;(August):4279-4292. doi:10.1002/sim.
